# Adverse outcomes of sarcopenic obesity in older adults: a scoping review

**DOI:** 10.3389/fnut.2026.1917374

**Published:** 2026-09-14

**Authors:** Hui Chang, Yanan Zhao, Ying Tian, Liang Hao

**Affiliations:** 1 Department of Cardiology, Xi’an People’s Hospital (Xi’an Fourth Hospital), Xi’An, China; 2 Department of Gynecology, Xi’an People’s Hospital (Xi’an Fourth Hospital), Xi’An, China; 3 Department of Surgery, Xi’an People’s Hospital (Xi’an Fourth Hospital), Xi’An, China

**Keywords:** adverse health outcomes, nutrition, older adults, sarcopenic obesity, scoping review

## Abstract

**Introduction:**

Sarcopenic obesity (SO) has been shown to have a substantial impact on health. However, the current understanding of adverse outcomes associated with SO is incomplete.

**Methods:**

A scoping review of the literature in PubMed, Web of Science, the Cochrane Library, the Cumulative Index to Nursing and Allied Health Literature (CINAHL), Embase, China National Knowledge Infrastructure (CNKI), and Wanfang Data was conducted from inception to 5 September 2025 to identify articles on adverse outcomes associated with SO in older adults.

**Results:**

A total of 4,954 studies met the screening criteria, and 21 studies met the inclusion criteria. These studies reported 11 adverse outcomes related to SO across four domains. (1) Impaired physical function and independence domains included a decline in instrumental activities of daily living (IADL; three studies), a decline in ADL (three studies), impaired body function (two studies), and disability (one study). (2) Physical health domains included cardiovascular disease (three studies), metabolic syndrome (one study), and mortality (nine studies). (3) Safety-related incident domains included fractures (two studies) and falls (two studies). (4) Neuropsychological and frailty status domains included depression (two studies) and frailty (one study).

**Conclusion:**

This scoping review suggests that SO is longitudinally associated with a range of adverse health outcomes. However, due to the small number of studies and limited sample sizes, more high-quality studies are necessary to further explore the longitudinal relationships between SO and adverse health outcomes.

## 1 Introduction

The global population is aging rapidly. As of 2019, there were 1 billion older adults worldwide, a number projected to reach 2.1 billion by 2050 (1). In China, which is experiencing profound aging, there were 310 million people aged 60 years and older in 2024, representing 22.0% of its total population (2). Among the various geriatric health issues, sarcopenia is a common geriatric syndrome characterized by age-related loss of muscle mass, decreased muscle strength, and reduced physical function (3). Recently, the 2025 Asian Working Group for Sarcopenia (AWGS) consensus updated its diagnostic framework, simplifying the criteria to require both low muscle mass and low muscle strength, although the role of physical performance remains debated (4). Studies indicate that the prevalence of sarcopenia among older adults worldwide ranges between 10 and 27%, and sarcopenia is closely associated with adverse health outcomes such as increased mortality, functional impairment, and falls (5). In addition, the aging process is often accompanied by the accumulation of body fat, and obesity itself is a significant risk factor for cardiovascular diseases and metabolic abnormalities (6). Research has found that sarcopenia often coexists with an increase in adipose tissue. The convergence of these two prevalent conditions—sarcopenia and obesity—has given rise to a new health concern known as sarcopenic obesity (SO) (7, 8).

SO is a clinical condition characterized by the simultaneous presence of obesity and sarcopenia (9). Its global prevalence in older adults is approximately 11% (10). Sarcopenia and obesity interact synergistically. Sarcopenia leads to a decline in the basal metabolic rate and reduced energy expenditure, thereby resulting in fat accumulation. Conversely, obesity exacerbates the loss of muscle mass and muscle strength through metabolic disturbances such as insulin resistance and chronic low-grade inflammation. Under the dual metabolic stress of these two conditions, a vicious cycle may develop, thereby collectively impairing human health (11). Studies have shown that individuals with SO face a significantly increased risk of complications such as disability, falls, frailty, and cardiometabolic diseases, which severely impact their quality of life and markedly elevate the rates of disability and all-cause mortality (11–14). Furthermore, SO contributes to higher rates of hospital readmission and increased medical costs, imposing a substantial economic burden on families, society, and the healthcare system.

Given the high prevalence of SO among older adults, understanding and evaluating its impact as a risk factor for other diseases is a public health priority. Recently, Minnetti et al. (15) conducted a systematic review that comprehensively synthesized the research evidence on adverse outcomes associated with sarcopenic obesity and applied the Sarcopenic Obesity Global Leadership Initiative algorithm to classify clinical outcomes. This study has significantly advanced the field. However, this review included a broader adult population and did not specifically target older adults. Moreover, its methodological approach was designed for evidence synthesis rather than evidence mapping or gap identification. In contrast, this scoping review specifically targets the older adult population and systematically synthesizes the available longitudinal evidence following the Arksey and O’Malley scoping review framework. It provides a domain-specific classification of outcomes tailored to geriatric clinical practice while clearly delineating areas with sufficient evidence from those with evident gaps, thereby informing future research priorities.

Therefore, this study conducted a scoping review of the literature on SO in older adults, aiming to systematically map SO-related adverse health outcomes. This review provides a theoretical foundation for mitigating the effects of these adverse health outcomes on the functional performance in older adults and promoting healthy aging.

## 2 Methods

This scoping review was conducted using a five-stage methodological framework developed by Arksey and O’Malley. The process involved the following stages: (1) formulating the research question; (2) searching for relevant literature; (3) applying predefined eligibility criteria to select studies; (4) extracting and charting the data; and (5) collating, synthesizing, and presenting the findings.

### 2.1 Identifying the research question

This scoping review aims to comprehensively map the adverse outcomes associated with SO in older adults. The research question was structured using the Population, Concept, and Context (PCC) framework recommended by the JBI guidance for scoping reviews. The population comprised older adults (≥60 years), the concept encompassed SO and its relationship with adverse health outcomes, and the context included community, outpatient, primary care, nursing homes, and hospital settings.

### 2.2 Identifying relevant studies

Our literature search was conducted in two key phases. First, we used an iterative process to develop the search strategy. We performed preliminary searches in PubMed using a combination of Medical Subject Headings (MeSH) and free-text terms to identify the most relevant keywords. This preliminary strategy was then reviewed and finalized by our entire research team. Second, the finalized search string was subsequently executed across seven electronic databases: PubMed, Embase, Web of Science, the Cochrane Central Register of Controlled Trials, the Cumulative Index to Nursing and Allied Health Literature (CINAHL), the China National Knowledge Infrastructure (CNKI), and Wanfang Data. The search strategy combined the search terms for the population—“aged,” “elderly,” “older,” and “geriatric”—and the condition—“sarcopenic obesity.” No date restrictions were applied, with searches covering all records from the inception of each database to 5 September 2025. The complete search syntax for all databases is provided in Supplementary Table S1.

### 2.3 Eligibility criteria for study selection

#### 2.3.1 Eligibility criteria

Studies were included if they met the following criteria: (1) Participants (P): studies including participants aged 60 years and older; (2) Concept (C): studies reporting the relationship between SO and adverse outcomes; and (3) Context (C): studies conducted in community, outpatient, primary care, nursing home, or hospital settings.

The exclusion criteria were as follows: (1) non-English or Chinese language publications; (2) duplicate publications; and (3) cross-sectional studies, case reports, case series, or ecological studies.

Cross-sectional studies were excluded because this review aimed to examine the longitudinal relationship between SO and adverse outcomes, which requires temporally ordered evidence that cross-sectional designs cannot provide.

#### 2.3.2 Study selection

The screening process was conducted independently by two reviewers (HC and YNZ). Initially, all retrieved records underwent title and abstract screening against the predefined eligibility criteria. Records that appeared to meet the criteria were advanced to the second stage for full-text assessment. In this stage, the same two reviewers independently evaluated the complete articles using the same criteria. Any discrepancies arising at either stage were resolved through discussion or, if necessary, by adjudication by a third senior reviewer (LH).

### 2.4 Charting the data

Relevant information was organized and extracted using a data extraction table designed for this study. The review panel examined this table, which included author information, year of publication, country, study type, assessment tool, and specific manifestations of adverse outcomes.

### 2.5 Collating, summarizing, and reporting the results

The author categorized the publications according to their study objectives. A descriptive summary was created for each adverse outcome. Following the recommendations of Arksey and O’Malley for scoping reviews, we did not critically appraise individual studies, as this review aimed to summarize the existing literature and identify knowledge gaps.

## 3 Results

### 3.1 Literature search results

The literature search initially yielded 4,954 records. After removing 1,552 duplicates, 3,402 unique records underwent title and abstract screening. At this stage, 3,316 records were excluded because they did not meet the eligibility criteria, leaving 86 articles for full-text retrieval and assessment. Following a detailed full-text review, an additional 65 articles were excluded, resulting in the final inclusion of 21 studies in this scoping review. The complete flow of the study selection process is detailed in Figure 1.

**Figure 1:**
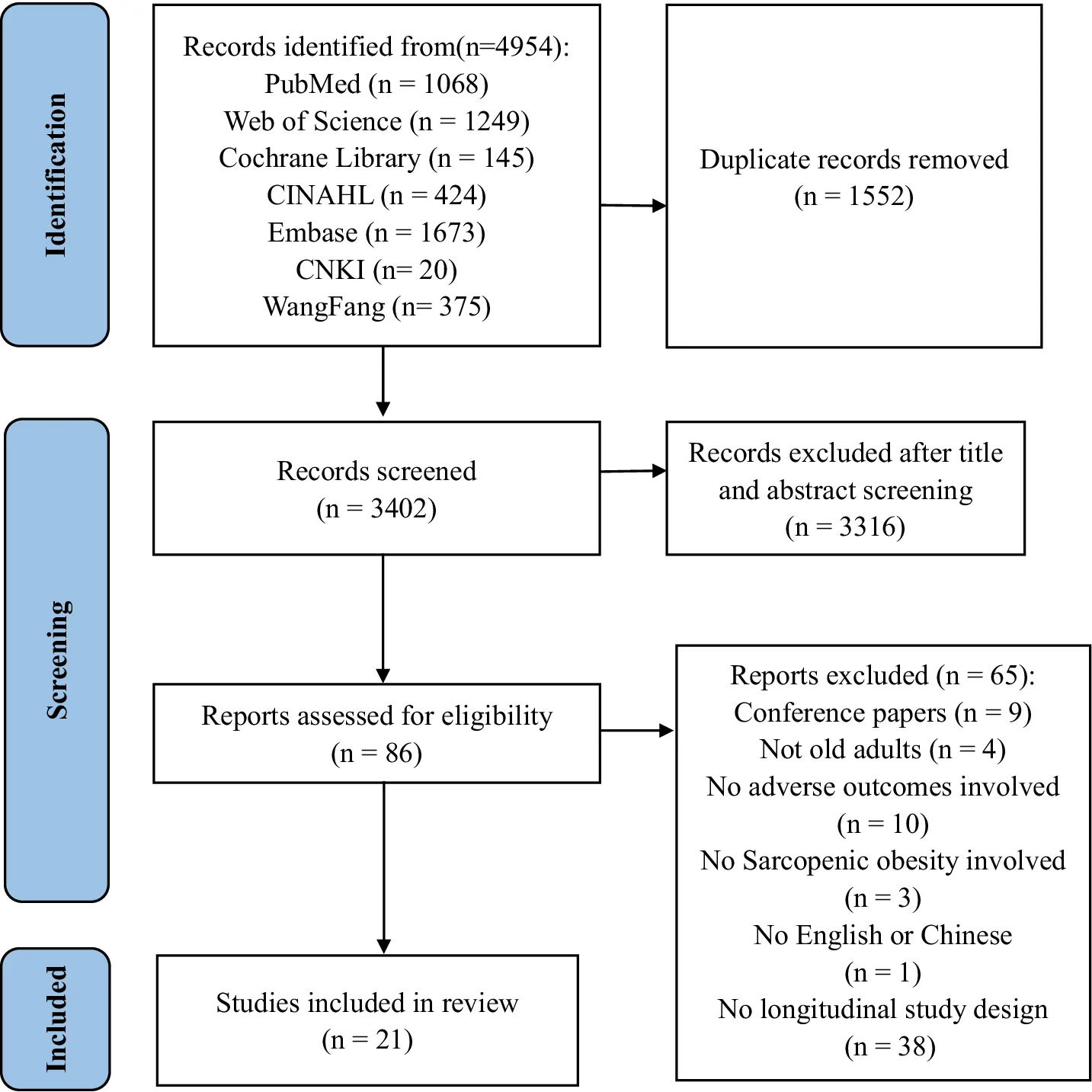
Flow diagram of the study selection and inclusion process.

### 3.2 Characteristics of the included studies

The 21 studies included in the review, published between 2004 and 2025, encompassed a total of 33,511 participants. Sample sizes varied widely, ranging from a minimum of 113 to a maximum of 4,321 participants. The follow-up periods of these longitudinal studies ranged from 3 months to 17.8 years, with two studies having follow-up periods of less than 1 year, six studies having follow-up periods of 1–3 years, and 13 studies having follow-up periods of more than 3 years. These studies were conducted across 10 countries: China, the United States, Canada, South Korea, the United Kingdom, Australia, the Netherlands, Sweden, Türkiye, and Japan. In terms of setting, the majority of studies were conducted in community settings (57.14%), followed by hospitals (33.33%), while nursing homes and outpatient clinics each accounted for 4.76%. A detailed summary of the study characteristics is presented in Table 1.

**Table 1:** Characteristics of included studies.

Authors, year	Country	Study design	Follow up time	Total sample	Age	Setting	SO assessment	Adverse outcomes
Baumgartner et al. (2004) (12)	USA	Longitudinal cohort study	1–8 years	451	≥60	Community	*Sarcopenia:* subjects were classified as sarcopenic if their relative skeletal muscle mass was 2 SD.	IADL decline
							*Obesity:* subjects were classified as obese if their PBF was above the 60th percentile of the study sample.	
Stephen et al. (2009) (29)	Canada	Longitudinal study	1–10 years	3,366	≥65	Community	*Sarcopenia:* sarcopenia was classified using two different approaches based on either skeletal muscle mass or skeletal muscle strength. Whole-body skeletal muscle mass was estimated using BIA. Muscle strength: maximal dominant hand grip strength was measured 3 times using a Jamar dynamometer and averaged to the nearest kg;	Cardiovascular disease
							*Obesity:* WC.	
Atkins et al. (2014) (13)	UK	Prospective cohort study	Mean 11.3 years	4,111	≥60	Community	*Sarcopenia:* midarm muscle circumference was used as a marker of muscle mass and was calculated as mid upper arm circumference (cm)–0.3142*9 triceps skinfold thickness (mm);	Mortality
							*Obesity:* WC.	
Hamer et al. (2015) (31)	UK	Longitudinal cohort study	1–6 years	3,862	≥60	Community	*SO:* SO was defined as obese individuals (BMI> = 30 kg/m^2^) in the lowest tertile of sex-specific grip strength (<35.3 kg men; <19.6 kg women).	Depression
Scott et al. (2016) (24)	Australia	Longitudinal cohort study	1–5 years	1,486	≥70	Community	*Sarcopenia:* the EWGSOP and FNIH Sarcopenia Project criteria were used to define sarcopenia in this study. The EWGSOP defines sarcopenia in men as ALM adjusted for height squared <7.25 kg/m^2^ combined with low hand-grip strength (<30 kg) and/or low gait speed (0.8 m/s). The FNIH derived ALM and hand-grip strength cut-points from nine different population-based studies and defines low lean mass criteria as ALM/BMI < 0.789 for men and hand-grip strength <26 kg.	Falls and fracture
							*Obesity:* Obesity was defined in this study as body fat percentage 30% because BF percentage is a more appropriate indicator of obesity than BMI.	
Hirani et al. (2017) (30)	Australia	Longitudinal cohort study	Median 7.0 years	1,685	≥70	Community	*Sarcopenia:* this study used the FNIH clinically relevant cut-points for low lean mass defined as ALM:BMI ratio less than 0.789 for men.	ADL decline, IADL decline, frailty
							*Obesity:* this study defined obesity as percent fat mass more than 30% according to recent definitions.	
Sanada et al. (2018) (14)	Japan	Longitudinal study	Mean 11.7 years	2,309	≥70	Community	*SO:* SO defined by three patterns of obesity indexes, PBF and WC and skeletal muscle index estimated by anthropometric measurements.	Mortality
Scott et al. (2018) (25)	Australia	Longitudinal cohort study	5 years	1705	≥70	Community	*Sarcopenia:* the EWGSOP criteria were used to define sarcopenia in this study. The EWGSOP defines sarcopenia in men as low ALM/height (<7.26 kg/m^2^) combined with low handgrip strength (<30 kg) and/or low gait speed (≤0.8 m/s).	Metabolic syndrome
							*Obesity:* obesity was defined in this study as BF percentage ≥30%.	
Atmis et al. (2019) (23)	Turkey	Longitudinal observational study	2 years	350	≥65	Hospital	*Sarcopenia:* sarcopenia was defined according to the EWGSOP. The presence of low muscle mass and presence of low muscle strength or low physical function were required to diagnose sarcopenia according to the EWGSOP criteria. Low muscle mass was classified as an SMI less than 9.2 kg/m^2^ in men and 7.4 kg/m^2^ in women. Muscle strength was assessed by HGS using a hand dynamometer.	Mortality
							*Obesity:* obesity was defined according to fat mass percentage.	
Kokkeler et al. (2019) (32)	Netherlands	Longitudinal cohort study	2 years	506	≥65	Hospital	*SO:* SO was measured by combining measures of maximum HGS and abdominal obesity (WC).	Depression
von Berens et al. (2020) (26)	Sweden	Longitudinal cohort study	10 years	521	≥75	Community	*Sarcopenia:* the updated EWGSOP2 definition advocates the use of reduced chair-stand capacity (time to perform five repeated chair stands >15 s) or reduced grip strength (<16 kg for women and<27 kg for men) in combination with low muscle mass.	Mortality
							*Obesity:* obesity to define obesity, any of three measures of obesity was used, i.e., BMI ≥ 30 kg/m2, fat mass>42% (women) and>30% (men), or waist circumference≥88 cm (women) and≥102 cm (men). If any of the obesity criteria were fulfilled, the individual was defined as having obesity.	
Chuan et al. (2022) (16)	China	Longitudinal cohort study	Mean 3.46 years	386	≥60	Hospital	*Sarcopenia:* Sarcopenia was defined as low muscle mass plus low muscle strength and/or low physical performance according to the AWGS criteria established in 2019. Low muscle mass was defined as SMI < 7.0 kg/m^2^ in males and <5.4 kg/m^2^ in females as measured by DXA. Low muscle strength was defined as handgrip strength <28 kg in males and <18 kg in females; the criterion for low physical performance was a 6-m gait speed <1.0 m/s.	Mortality, fracture incident, cardiovascular disease
							*Obesity:* adiposity included an excess of whole-body fat or abdominal fat mass. In this study, excessive whole-body fat was defined as meeting one of three different criteria: the Working Group on Obesity in China guideline (BMI of 28 kg/m^2^ or more), WHO Western Pacific Region guideline (BMI of 25 kg/m^2^ or above), and the WHO recommendation (BF% >25% for men or 35% for women). Moreover, abdominal obesity was defined in two ways as follows: VFA ≥ 100 cm^2^ according to the Chinese Medical Association guidelines, and AF higher than the sex-specific median in the present cohort (>1.69 kg for men, >1.75 kg for women).	
Saito et al. (2022) (17)	Japan	Longitudinal cohort study	1 year	779	≥65	Hospital	*Sarcopenia:* sarcopenia was defined according to the AWGS criteria. Following the AWGS criteria, participants were diagnosed with sarcopenia when low muscle strength or physical performance coexisted with low skeletal muscle mass. We defined low muscle strength as handgrip strength <26 kg for men and <18 kg for women; low physical performance was defined as a 4-m walk time ≤0.8 m/s for both sexes.	Mortality, impaired body function
							*Obesity:* obesity was defined based on the percentage of BFM obtained from the BIA. Cut-off points for obesity were 38 and 27% FM for women and men, respectively.	
Morikawa et al. (2023) (11)	Japan	Longitudinal cohort study	5 years	4,197	≥65	Community	*Sarcopenia:* sarcopenia was identified using AWGS 2019 algorithm, which consisted of 3 domains (muscle strength, physical performance, and muscle mass). Muscle strength was assessed using the HGS. Participants were considered having low muscle mass strength if HGS was<28 kg for men and <18 kg for women. Physical performance was assessed by gait speed (m/s), 5 times chair stand test, and Short Physical Performance Battery. The skeletal muscle mass was converted into skeletal muscle mass index by dividing the muscle mass by the square of the participant’s height (kg/m^2^). If skeletal muscle mass was lower than cut-off values (<7.0 kg/m^2^ for men and<5.7 kg/m^2^ for women), the participants were categorized as having low muscle mass.	Disability
							*Obesity:* obesity was determined when body fat percentage was 25% or VF was 100cm^2^ for either sex.	
Ulugerger et al. (2023) (21)	Turkey	Longitudinal cohort study	5 years	175	≥65	Outpatient clinic	*Sarcopenia:* Sarcopenia consisted of 3 domains (muscle strength, physical performance, and muscle mass). SMMI adjusted for height was calculated as skeletal muscle mass (kg)/height^2^ (m^2^). SMMI was defined as sarcopenia when it was <7.4 kg/m^2^ in women and <9.2 kg/ m^2^ in men with adjustment for height. Muscle strength was assessed by HGS. This study defined low HGS thresholds according to the European consensus (<16 kg and <27 kg for females and males, respectively). Physical performance was evaluated by 6-m gait speed. Walking speed was determined as usual when the test result was ≥0.8 m/s.	Mortality
							*Obesity:* obesity is defined as a BMI ≥ 30 kg/m^2^.	
Chen et al. (2025) (18)	China	Longitudinal cohort study	2 years	695	≥60	Nursing home	*Sarcopenia:* sarcopenia diagnosis is based on the 2019 consensus of the AWGS, requiring concurrent presence of low muscle mass and low muscle strength and/or low physical function. Low muscle mass is defined as a SMI < 7 kg/m^2^ for men and <5.7 kg/m^2^ for women. Muscle strength is measured by HGS, defined as <28 kg for men and <18 kg for women. Physical function is assessed via 4-meter walking speed and the 5-repetition sit-to-stand test; walking speed <1 m/s or sit-to-stand time ≥12 s indicates low physical function.	Mortality
							*Obesity:* six distinct obesity indicators were employed: WC, WHR, WHTR, BMI, VFA, and PBF. The diagnostic cut-off points for each indicator are as follows: WC: ≥90 cm for males, ≥80 cm for females; WHR: ≥0.90 for males, ≥0.85 for females; WHTR: ≥0.5; BMI: ≥28 kg/m^2^; VFA: ≥100 cm^2^; PBF: ≥20% for males, ≥30% for females.	
Yoon et al. (2025) (19)	Korea	Longitudinal cohort study	Mean 17.8 months	317	≥65	Hospital	*Sarcopenia*: sarcopenia was defined as a low ASM combined with a low muscle strength, low physical performance, or both. Low skeletal muscle mass was defined as height-adjusted ASM less than 7.0 kg/m^2^ in men and less than 5.4 kg/m^2^ in women. Low physical performance was defined as gait speed less than 1.0 m/s for both men and women, and low muscle strength was defined as handgrip strength less than 28 kg in men and less than 18 kg in women.	Cardiovascular disease
							*Obesity:* visceral obesity was defined as visceral adipose tissue area of 134.6 cm^2^ or greater in men and 91.1 cm^2^ or greater in women.	
Yu et al. (2025) (22)	China	Longitudinal cohort study	10.9 years	4,321	≥60	Community	*Sarcopenia:* the ESPEN/EASO consensus criteria for Asian populations, define sarcopenia as a low SMM/BW (<38.2% for men and <32.2% for women) and/or a low ALM/ BW (<32.5% for men and<25.7% for women) with a low HGS (<28 kg for men and <18 kg for women).	Cardiovascular disease
							*Obesity:* obesity was defined as a high FM/BW (>29% for men and >41% for women).	
Zong et al. (2024) (20)	China	Longitudinal cohort study	3 months	113	≥65	Hospital	*Sarcopenia:* sarcopenia was diagnosed through a reduction in either ASMI and HGS or ASMI and 6-meter gait speed. Based on the AWGS, ASMI less than 7.0 kg/m^2^ (male) or 5.7 kg/m^2^ (female) was defined as decreased muscle mass. Diminished muscle strength is defined by a HGS measurement of less than 28 kg for males or 18 kg for females. Additionally, a 6-meter gait speed of less than 1.0 m/s was considered indicative of reduced muscular endurance.	Mortality
							*Obesity:* PBF exceeding 25% (male) or 35% (female) was considered obese status.	
Scott et al. (2023) (27)	Australia	Longitudinal cohort study	1 year	1,416	≥70	Community	*Sarcopenia:* Sarcopenia was defined using the EWGSOP 2 definition. Probable sarcopenia: low HGS (<27 kg) or high chair-stands time (>15 s); Confirmed sarcopenia: low HGS or high chair-stands time with ALM/H < 7.0 kg/m^2^. According to the ESPEN-EASO criteria, sarcopenic obesity was diagnosed if both altered skeletal muscle function (low HGS < 27 kg or chair-stands time ≥17 s) and altered body composition (low ALM/W < 25.7% and high fat mass ≥26%) were present.	ADL decline, IADL decline, impaired body function, fall
							*Obesity:* BMI (≥30 kg/m^2^) or WC (≥90 cm).	
Yoshimura et al. (2022) (33)	Japan	Longitudinal cohort study	Median 3–5 months	760	≥65	Hospital	*Sarcopenia:* Low HGS (<28 kg for men, <18 kg for women) and low SMM/W (<31.5% for men, <22.1% for women);	ADL
							*Obesity:* High FM% (>29.7% for men, >37.2% for women).	

AFM, android fat mass; ALM, appendicular lean mass; ASM, appendicular skeletal muscle mass; ASMI, appendicular skeletal muscle mass index; AWGS, Asian Working Group for Sarcopenia; BFM, body fat mass; BIA, bio-impedance analysis; BF%, body fat percentage; BMI, body mass index; BW, body weight; DXA, dual-energy X-ray absorptiometry; EASO, European Association for the Study of Obesity; ESPEN, European Society for Clinical Nutrition and Metabolism; EWGSOP, European Working Group on Sarcopenia in Older People; FNIH, Foundation for the National Institutes of Health; FM%, fat mass percentage; HGS, handgrip strength; PBF, percentage body fat; SMI, skeletal muscle index; SMM, skeletal muscle mass; VFA, visceral fat area; WC, waist circumference; WHR, waist–hip ratio; WHTR, waist–height ratio.

### 3.3 Measures of sarcopenic obesity

According to the “Definition and Diagnostic Criteria for Sarcopenic Obesity” (9), jointly released by the European Society for Clinical Nutrition and Metabolism (ESPEN) and the European Association for the Study of Obesity (EASO), scholars from both domestic and international backgrounds agree that an individual is considered to have SO when they are at a dual risk of obesity and sarcopenia.

For assessing sarcopenia, seven studies utilized the definition established by the Asian Working Group for Sarcopenia (AWGS) (11, 16–21). According to the AWGS consensus, sarcopenia is defined as low skeletal muscle mass accompanied by low muscle strength and/or low physical performance. A majority of studies measured muscle mass using dual-energy X-ray absorptiometry (DXA) (11, 16, 18, 20). Low muscle mass was defined as a skeletal muscle index (SMI) of <7.0 kg/m^2^ in men and <5.4 kg/m^2^ in women. Muscle strength was predominantly measured using handgrip strength, with low muscle strength defined as <28 kg in men and <18 kg in women (16–22). Low physical performance was defined as a 6-m walking speed of <1.0 m/s (11, 16, 19, 21) or a 4-m walking speed of <0.8 m/s (17, 18, 23, 24). In total, five studies used the definition of sarcopenia established by the European Working Group on Sarcopenia in Older People (EWGSOP) to define sarcopenia (23–27). A total of two studies applied the consensus criteria established by the European Society for Clinical Nutrition and Metabolism (ESPEN) and the European Association for the Study of Obesity (EASO) to define sarcopenia (22, 28). In addition, four studies defined sarcopenia based on muscle mass (12, 14, 29, 30), two studies defined it based on muscle strength (31, 32), and one study defined it based on mid-arm muscle circumference (13). Regarding the assessment of obesity, 12 studies defined obesity using body fat percentage (11, 12, 14, 17, 18, 20, 22–26, 30), five studies used waist circumference (13, 18, 26, 29, 32), three studies used visceral fat area (16, 18, 19), and five studies used BMI (14, 16, 21, 26, 31).

Notably, the diagnostic criteria for SO varied substantially across the 21 included studies. Of these, 14 (66.7%) studies were published before 2022. Sarcopenia was defined using six different approaches: the AWGS criteria (*n* = 7), the EWGSOP criteria (*n* = 5), the ESPEN–EASO consensus criteria (*n* = 2), muscle mass alone (*n* = 4), muscle strength alone (*n* = 2), and mid-arm muscle circumference (*n* = 1). Obesity was defined using four different measures: Body fat percentage (*n* = 12), waist circumference (*n* = 5), visceral fat area (*n* = 3), and BMI (*n* = 5).

### 3.4 Adverse health outcomes predicted by sarcopenic obesity

The summary of studies on adverse outcomes associated with SO in older adults, as presented in Supplementary Table S2, included findings from 21 studies. These studies reported a total of 11 adverse outcomes, with individual studies documenting between 1 and 4 adverse effects each. The most frequently reported adverse outcome was mortality (13, 14, 16–18, 20, 21, 23, 26), followed by cardiovascular disease (19, 22, 29), IADL decline (12, 27, 30), ADL decline (27, 30, 33), depression (31, 32), fractures (16, 24), falls (24, 27), and impaired body function (17, 27). The following adverse outcomes were each reported in only one study: metabolic syndrome (25), frailty (30), and disability (11). We will provide a clearer and more insightful interpretation of the results by conceptually categorizing adverse outcomes and identifying common themes and patterns. The above adverse outcomes can be categorized into the following four core domains:

#### 3.4.1 Impaired physical function and independence

The impaired physical function and independence domains primarily included IADL decline (12, 27, 30), ADL decline (27, 30, 33), impaired body function (17, 27), and disability (11). The studies found that patients with SO exhibited a significantly increased risk of IADL impairment. Baumgartner et al. (12) found that, among patients with SO, the relative risk of new-onset IADL decline was 2.63 (95% confidence interval: 1.19–5.85). Hirani et al. (30) found that SO was significantly associated with IADL impairment in community-dwelling older men (OR = 1.36, 95% CI: 1.05–1.76). Furthermore, the study also indicated a significant association between SO and ADL impairment in community-dwelling older men (OR = 1.58, 95% CI: 1.12–2.24) (27, 30, 33). Two studies assessed physical performance in older adults using measures such as walking speed and the Short Physical Performance Battery (17, 27). The findings indicated that SO could predict declines in physical function in older adults. A five-year longitudinal study conducted in Japan defined disability based on certification for long-term care under public insurance. This study indicated that patients with SO face a significantly increased risk of disability (HR = 2.48, 95% CI 2.24–2.75) (11).

#### 3.4.2 Physical health status

Physical health domains included cardiovascular disease (19, 22, 29), metabolic syndrome (25), and mortality (13, 14, 16–18, 20, 21, 23, 26).

Three studies reported adverse outcomes related to cardiovascular disease (19, 22, 29). Stephen et al. indicated that muscle strength may be more important than muscle mass in protecting against cardiovascular disease. Their study found that, when SO was defined by waist circumference and muscle strength, the risk of cardiovascular disease increased by 23% in the SO group. However, when waist circumference and muscle mass were used as criteria, the risk of cardiovascular disease did not significantly increase in the SO group (29). Yoon et al. indicated that patients with SO had the highest risk of cardiovascular disease (HR = 6.74, 95% CI: 1.81–25.16) (19). Yu et al. (22) also reported similar findings.

There was one article that reported adverse outcomes related to metabolic syndrome (25). A study in Australia found that older men with SO had more than twice the risk of developing metabolic syndrome (25).

Nine articles reported adverse outcomes related to mortality (13, 14, 16–18, 20, 21, 23, 26). Five studies indicated that SO is independently associated with all-cause mortality in older adults. These studies reported that the mortality risk among sarcopenic obese older adults was 1.41–6.54 times higher than that among non-obese individuals (13, 17, 20, 21, 23). In addition, one study indicated that, in female patients aged 75 years, SO was significantly associated with a 10-year mortality risk (HR = 3.25, 95% CI 1.2–8.9). However, this association was not statistically significant in male patients (26). Furthermore, different studies employed varying definitions of obesity within the diagnosis of sarcopenic obesity, and the significance of the association between SO and mortality also exhibited certain discrepancies. Studies by Sanada et al. (14) indicated that, when SO was defined by waist circumference (HR = 1.19, 95% CI 1.02–1.38) and BMI (HR = 1.15, 95% CI 1.00–1.33), it was significantly associated with an increased risk of mortality in patients. However, SO defined by body fat percentage was not associated with patient mortality risk (14). A study by Chuan et al. indicated that SO defined by body fat percentage (HR = 2.94, 95% CI 1.25–6.92), BMI (HR = 13.38, 95% CI 2.82–63.48), visceral obesity (HR = 4.60, 95% CI 1.25–16.94), and abdominal fat mass (HR = 3.77, 95% CI 1.38–10.28) was significantly associated with patient mortality (16). In addition, the study by Chen et al. (18) demonstrated that SO defined by body fat percentage was significantly associated with patient mortality. In contrast, definitions based on waist circumference, waist–hip ratio, and waist–height ratio showed no significant association with mortality risk.

#### 3.4.3 Safety-related incidents

The safety-related incident domains included fractures (16, 24) and falls (24, 27). Two studies reported falls as an adverse outcome (24, 27). Scott et al. (24) noted that SO defined according to the EWGSOP was significantly associated with the incidence of falls over 2 years in older adults, whereas SO defined according to the Foundation for the National Institutes of Health (FNIH) was not associated with fall incidence in older adults. Similarly, ESPEN–EASO-defined sarcopenic obesity was also associated with higher 12-month incident fall rates (OR = 2.03; 95% CI = 1.23–3.36) (27). In total, two studies reported fractures as an adverse outcome (16, 24). Scott et al. reported that older men with sarcopenic obesity, as defined by the FNIH, had an increased risk of fracture over 6 years (24). Chuan et al. (16) also noted that SO increases the incidence of fractures in older adults.

#### 3.4.4 Neuropsychological and frailty status

The neuropsychological and frailty status domains included depression (31, 32) and frailty (30). There were two studies that reported depression as an adverse outcome (31, 32). Both studies indicated that SO can predict the onset of depression in older adults. One study reported frailty as an adverse outcome (30). Hirani et al. (30) showed that SO was associated with an increased risk of frailty in older male patients (OR = 2.00, 95% CI: 1.42, 2.82).

## 4 Discussion

### 4.1 Main findings

This study aimed to identify the adverse outcomes associated with SO in older adults. To broadly explore this emerging field, we reviewed longitudinal studies with follow-up periods ranging from months to years. A total of 11 adverse outcomes were reported across the 21 studies included in this review. The most frequently reported adverse outcome was mortality, followed by cardiovascular disease, IADL decline, ADL decline, depression, fractures, falls, and impaired body function. The following adverse outcomes were each reported in only one study: metabolic syndrome, frailty, and disability. For adverse outcomes reported in only one study, further studies are needed to verify the reliability of the results.

### 4.2 Adverse outcomes of sarcopenic obesity

#### 4.2.1 Impaired physical function and independence

This review found that SO was consistently associated with impaired physical function and loss of independence in older adults. IADL impairment was frequently reported as an adverse outcome (12, 27, 30), reflecting significant difficulties among older adults with SO in performing complex daily tasks such as shopping, cooking, and medication management. Declines in ADL have similarly been documented in three studies (27, 30, 33), further suggesting that, as sarcopenic obesity progresses, older adults with SO may progressively lose fundamental self-care capabilities, such as dressing and grooming. Two interconnected pathways drive this process. On the one hand, sarcopenia directly impairs limb mobility, balance, and exercise endurance in older adults by reducing muscle mass and strength (34). On the other hand, the additional weight from obesity places further strain on already weakened muscles, creating a vicious cycle: reduced muscle strength makes physical activity more difficult, which, in turn, accelerates muscle loss and functional decline (35). This clinically observable cycle does not exist in isolation. It is driven and sustained by underlying biological disturbances. Inflammatory responses and insulin resistance originating in adipose tissue can promote muscle protein breakdown and impair energy metabolism in skeletal muscle (7). In contrast, ectopic fat deposition within muscle further impairs muscle contraction quality and physical performance (36). Through these pathways, the mechanical cycle is reinforced at the tissue level, trapping patients with SO in a vicious cycle in which muscle loss, fat accumulation, and progressive functional decline mutually exacerbate one another. This decline in independence not only severely impacts the quality of life but also significantly increases the reliance on caregivers. Therefore, a tiered intervention strategy should be implemented based on the functional status of older adults.

#### 4.2.2 Physical health status

In the domain of physical health status, the main adverse health outcomes included cardiovascular disease (19, 22, 29), metabolic syndrome (25), and mortality (13, 14, 16–18, 20, 21, 23, 26).

Regarding cardiovascular diseases, several studies have explored the association between SO and the incidence of CVD; however, the findings remain inconsistent. Research by Yu et al. (22) and Yoon et al. (19) demonstrated that SO is a significant independent risk factor for CVD. Stephen et al. (29) indicated that, when SO was defined as an increased waist circumference combined with decreased muscle strength, the risk of cardiovascular disease in SO patients increased by 23%. However, when SO was defined using waist circumference and muscle mass, the risk of cardiovascular disease in the SO group did not show a significant increase (29). This finding is consistent with the EWGSOP2 perspective, which suggests that the impact of SO on cardiovascular health may be more closely related to declines in muscle strength rather than reductions in muscle mass alone (37). This may be because muscle strength is a key indicator of muscle function, and its decline can exacerbate inflammatory responses (38). Interleukin 6 (IL-6), tumor necrosis factor alpha (TNF-α), and C-reactive protein (CRP) have been reported as serum inflammatory markers of heart failure, and elevated levels of these markers are also associated with decreased muscle strength in older adults (39). Therefore, the association between SO and cardiovascular disease likely stems from increased inflammation induced by reduced muscle strength, which, in turn, elevates the levels of inflammatory markers, such as IL-6, TNF-α, and CRP, in the blood, ultimately increasing the risk of cardiovascular disease.

Regarding metabolic syndrome, one study consistently demonstrated that SO can significantly predict the onset of metabolic syndrome in older adults (25). A meta-analysis by Khadra et al. (40) revealed that the risk of developing type 2 diabetes in individuals with SO was 38% higher than those without SO. The association between SO and metabolic syndrome is primarily driven by adipose tissue dysfunction. Lipid accumulation triggers chronic inflammation and disrupts the secretion of adipokines, thereby promoting insulin resistance. Since skeletal muscle accounts for approximately 80% of insulin-mediated glucose uptake, this state of insulin resistance can be further exacerbated by a reduction in muscle mass, creating a vicious cycle (41). Metabolic disturbances accelerate muscle breakdown, and muscle loss, in turn, exacerbates glucose metabolism abnormalities (42). Therefore, excessive adipose tissue, rather than a simple reduction in muscle mass, is the primary driver of metabolic syndrome risk in patients with SO (43).

Furthermore, multiple studies have explored the relationship between SO and mortality (13, 17, 20, 21, 23), with results indicating that individuals with SO have a higher risk of death. A meta-analysis also concluded that the mortality rate among SO patients is 21% higher than that among non-SO individuals (44). However, some studies did not identify SO as an independent predictor of mortality risk (14, 16). These discrepancies may be attributed to inconsistencies in the diagnostic criteria and measurement tools used to define SO across studies. When interpreting the adverse health outcomes associated with SO, it is also necessary to consider the controversial phenomenon known as the “obesity paradox.” In 2002, Gruberg et al. (45) first observed that, among patients with coronary heart disease, those who were overweight or obese actually had a better prognosis than those with normal weight. If simple obesity has a survival-protective effect under certain circumstances, the risks associated with SO may not simply reflect the sum of the two factors but may instead involve a more complex interaction. In the case of SO, muscle loss depletes protein reserves and impairs the ability to recover from stress, while obesity-related chronic inflammation accelerates muscle breakdown, thereby offsetting any potential protective effects of simple obesity (7). This also explains why using BMI to define obesity tends to confound simple obesity with SO, thereby diluting the true association. In contrast, when body fat percentage or visceral fat area is used, the association between SO and mortality is more consistent (14, 16). Future studies should conduct stratified analyses based on obesity phenotypes to determine whether the obesity paradox holds true in the context of SO.

#### 4.2.3 Safety-related incidents

In the domain of safety-related incidents, the main adverse health outcomes included fractures (16, 24) and falls (24, 27). Regarding falls, two studies demonstrated that SO is a significant predictor of falls in older adults. Yu et al. indicated that the coexistence of sarcopenia and obesity may have synergistic adverse effects on balance, postural control, and overall physical function, thereby increasing the risk of falls (46). Other studies have also shown that muscle loss and excessive fat deposition impair physical function, mobility, and neuromuscular coordination (47). In terms of fractures, the studies included in this review also confirmed SO as a significant predictor of fractures (16, 24). On the one hand, declines in muscle mass and strength weaken mechanical loading stimulation of the bones and reduce the protective response capacity during falls (16). On the other hand, obesity-related metabolic abnormalities may adversely affect bone quality. Therefore, individuals with SO may face a dual threat of increased susceptibility to falls and heightened bone fragility, leading to an amplified risk of fractures (28).

#### 4.2.4 Neuropsychological and frailty status

Neuropsychological and frailty status domains included depression (31, 32) and frailty (30). First, SO significantly increases the risk of depression in older adults. A systematic review by Pilati et al. (48) reached a similar conclusion. On the one hand, the decline in physical function, reduced activity endurance, and limitations in daily activities caused by SO may lead to decreased social participation, diminished self-worth, and social isolation, all of which are common psychosocial triggers for depression (49). On the other hand, from a biological perspective, SO is often accompanied by a chronic low-grade inflammatory state. Pro-inflammatory cytokines, such as IL-6 and TNF-α, can affect neurotransmitter metabolism and neuroendocrine function, directly contributing to the pathogenesis of depression (48, 49). Thus, the relationship between SO and depression can be viewed as a vicious cycle formed by physiological dysfunction, social and behavioral withdrawal, and neuroendocrine–immune dysregulation. This finding suggests that routine depression screening should be implemented for older adults with SO in clinical practice, along with integrated intervention strategies that address both physiological and psychological aspects.

Meanwhile, SO is also a key factor contributing to the increased risk of frailty in older adults. SO can be regarded as a core manifestation and a powerful driver of frailty at the level of body composition. The reduction in muscle mass and decline in strength correspond directly to the “decreased muscle strength” and “exhaustion” phenotypes of frailty. Excessive fat accumulation not only increases physiological load but also accelerates protein breakdown and impairs muscle mass and function through related metabolic disturbances, thereby exacerbating the depletion of an individual’s physiological reserves (50). This dual imbalance in muscle and adipose tissue collectively weakens the body’s ability to maintain internal homeostasis, making individuals with SO more susceptible to progressing into a state of frailty (30).

### 4.3 Follow-up duration and its implications for outcome interpretation

The 21 included studies exhibited considerable heterogeneity in follow-up durations, ranging from 3 months to 17.8 years. This wide temporal span has important implications for interpreting the observed associations between SO and adverse outcomes. Short-term studies (e.g., <1 year) are more likely to capture acute or subacute complications, such as falls or short-term functional decline following acute events, whereas long-term studies (e.g., ≥5 years) are better suited to detect slowly accumulating damage, including mortality and cardiovascular disease. Notably, the timing of outcome occurrence may also reflect distinct underlying mechanisms: Early outcomes may be driven predominantly by obesity-related mechanical burdens and inflammation, whereas later outcomes likely involve progressive muscle depletion and cumulative metabolic decompensation (51). In addition, the varying follow-up durations may contribute to discrepant findings across studies. For instance, a shorter observation window might underestimate mortality risk if the survival disadvantage associated with SO emerges only after several years, whereas a longer follow-up could introduce competing risks from age-related comorbidities (52). Despite these considerations, the limited number of studies and the inconsistent reporting of outcome timing across studies precluded a formal subgroup analysis by follow-up duration. Future research should systematically report time-to-event data and stratify analyses by follow-up period to clarify the temporal dynamics of SO-related adverse outcomes.

### 4.4 Strengths and limitations

This appears to be the first scoping review focused on the predictive role of SO for adverse outcomes in older adults, offering a consolidated evidence base to guide subsequent research. Nonetheless, this review has three main limitations. First, the wide range of follow-up durations (from 3 months to 11.7 years) poses a challenge to synthesizing a uniform predictive timeline. Second, the inclusion of studies conducted in diverse settings (e.g., community and hospital settings) may have influenced the generalizability of the findings, as contextual factors likely affect outcome manifestation. Third, the exclusion of cross-sectional studies, while methodologically justified by our focus on longitudinal predictive relationships, may have narrowed the evidence base and excluded valuable descriptive data. Finally, the 21 longitudinal studies included in this review exhibited significant heterogeneity in their diagnostic criteria for SO. This lack of consistency in criteria not only undermines comparability across studies but also affects the estimates of the effect of SO on clinical outcomes. These discrepancies likely stem from differences in the diagnostic criteria themselves rather than fluctuations in the true relationship between SO and outcomes. Although the ESPEN/EASO has published a consensus algorithm for SO diagnosis, and relevant academic organizations in China also issued a consensus in 2025 to promote diagnostic standardization, it will take time for these consensus guidelines to be widely adopted, and the heterogeneity observed in the current literature is unlikely to be resolved in the short term. Notably, 14 of the 21 studies (66.7%) were published prior to the release of the first unified ESPEN/EASO consensus in 2022. Ideally, subgroup analyses should be conducted based on diagnostic criteria or publication date to determine whether associations differ across different definitions. However, the limited number of studies within each definition-based subgroup precludes such analyses. Therefore, the evidence synthesized in this review should be interpreted with caution. Future systematic reviews and meta-analyses must first conduct rigorous stratification or subgroup analyses based on the diagnostic criteria of the included studies. At the same time, there is an urgent need for more large-scale, multicenter, longitudinal studies using uniform diagnostic criteria to provide a more robust evidence base for the causal relationship between SO and adverse outcomes.

## 5 Conclusion

This scoping review identified 11 adverse health outcomes associated with SO in older adults, including mortality, cardiovascular disease, functional decline (IADL/ADL), metabolic syndrome, depression, fracture, impaired physical function, falls, frailty, and disability. However, the evidence base remains uneven, with some outcomes supported by only one or two studies. Consequently, there is a clear need for future large-scale, multicenter longitudinal studies to quantify these associations robustly. The multiplicity of identified risks underscores the need for clinical and public health interventions that address the complex, multidimensional nature of aging with SO. By mapping this landscape, our study contributes to the foundational knowledge required for developing strategies to promote healthy aging and overall well-being in this vulnerable population.
