# Supplementary material for: Adverse outcomes of sarcopenic obesity in older adults: a scoping review
Source: Front Nutr. 2026 Sep 14;13:1917374. doi: 10.3389/fnut.2026.1917374 (PMC13616638; doi:10.3389/fnut.2026.1917374)
Supplement: Supplementary file 1 [file Table_1.DOCX]

**Table S1.** Search strategy

| **Total: 4954** | |
| --- | --- |
| 2025.9.5(Items found:1068) | |
| 1.Pubmed | |
| #1 | "Aged"[Mesh] OR "Geriatrics"[Mesh] |
| #2 | "aged"[Title/Abstract] OR "elderly"[Title/Abstract] OR "older"[Title/Abstract] OR "older people"[Title/Abstract] OR "older adults"[Title/Abstract] OR "senior*"[Title/Abstract] OR "geriatric*"[Title/Abstract] |
| #3 | #1 OR #2 |
| #4 | “sarcopenic obesity”[Title/Abstract] |
| #5 | #3 AND #4 |
| 2025.9.5(Items found:1249) | |
| 2. Web of science | |
| #1 | aged (Topic) OR elderly (Topic) OR older* (Topic) OR senior* (Topic) OR geriatric* (Topic) |
| #2 | "sarcopenic obesity" (Topic) |
| #8 | #1 AND #2 AND #6 AND #7 |
| 2025.9.5(Items found:145)  3.Chochrane Library | |
| #1 | MeSH descriptor: [Aged] explode all trees |
| #2 | (aged):ti,ab,kw OR (older*):ti,ab,kw OR (senior*):ti,ab,kw OR (elderly):ti,ab,kw |
| #3 | #1 OR #2 |
| #4 | (sarcopenic obesity):ti,ab,kw |
| #5 | #3 AND #4 |
| 2025.9.5(Items found:424) | |
| 4.CINAHL | |
| #1 | TI aged OR TI elderly OR TI older* OR TI senior* |
| #2 | AB aged OR AB elderly OR AB older* OR AB senior* |
| #3 | SU aged OR SU elderly OR SU older* OR SU senior* |
| #4 | #1 OR #2 OR #3 |
| #5 | TI sarcopenic obesity OR AB sarcopenic obesity OR SU sarcopenic obesity |
| #6 | #4 AND #5 |
| 2025.9.5(Items found:1673) | |
| 5. Embase | |
| #1 | 'aged'/exp |
| #2 | aged:ab,ti OR elderly:ab,ti OR older*:ab,ti OR senior*:ab,ti |
| #3 | #1 OR #2 |
| #4 | 'sarcopenic obesity':ab,ti |
| #5 | #3 AND #4 |
| 2025.9.5(Items found:20) | |
| 6.CNKI | |
| #1 | (主题=老年人) OR (主题=老人) |
| #2 | (主题=肌少性肥胖) OR (主题=肌肉减少性肥胖) |
| #3 | #1 AND #2 |
| 2025.9.5(Items found:375) | |
| 7.Wang Fang | |
| #1 | 主题:(老年人) or 主题:(老人) |
| #2 | 主题:(肌肉减少性肥胖) or 主题:(肌少性肥胖) |
| #3 | #1 AND #2 |

**Table S2 Four core domains for the adverse outcomes of SO.**

| **Core domains** | **Adverse outcomes** | **Results** |
| --- | --- | --- |
| **Impaired physical function and independence** | |  |
|  | IADL decline(1-3) | 1. Compared with other groups, the risk of newly developing self-reported IADL impairment was significantly increased by2.63 times (HR=2.63; 95% CI =1.19-5.85) in the SO group(1). 2. SO was significantly associated IADL disability (OR=1.32; 95% CI= 1.06-1.64)(2). 3. Patients with SO had a significantly higher likelihood of IADL disability than those without sarcopenia and who were not obese (OR = 2.17; 95% CI = 1.37-3.44)(3). |
|  | ADL decline(2-4) | 1. SO was significantly associated with ADL disability (OR=1.58; 95% CI=1.12-2.24)(2). 2. Patients with SO are significantly more likely to experience a decline in ADLs than those without sarcopenia who are not obese (OR = 4.88; 95% CI = 1.80-13.23)(3). 3. SO was independently and negatively associated with the functional independence measure in older men (coefficient = -0.117, *P* < 0.001) and women (coefficient = -0.048, *P* = 0.031)(4). |
|  | Impaired body function(3, 5) | 1. Patients with SO had significantly lower SPPB scores (coefficient=− 0.120; t-value=− 3.74; *P* < 0.001); Patients with sarcopenic obesity had significantly shorter 6MWD than those with non-sarcopenia/non-obesity after adjusting for age and sex (coefficient=− 77.42; t-value=− 3.61; *P* < 0.001)(5). 2. Older adults with SO exhibit lower handgrip strength (coefficient=− 5.87; 95% CI=-7.08-4.66) and slower gait speed (coefficient=− 0.11; 95% CI=-0.15- -0.08) compared to those without the condition(3). |
|  | Disability(6) | SO is significantly associated with disability in older adults (HR= 2.36; 95% CI = 1.53-3.63)(6). |
| **Physical health status** | Cardiovascular disease(7-9) | 1. When defining SO based on waist circumference and muscle strength, patients in the SO group had a 23% higher risk of cardiovascular disease compared with the normal group, although this association did not reach statistical significance (HR = 1.23; 95% CI = 0.99-1.54)(7). 2. Compared to patients without obesity, those with SO had the highest risk of cardiovascular disease (HR=6.74; 95% CI=1.81-25.16)(8). 3. Compared with participants without sarcopenia or obesity, those with SO had the highest risk of atrial fibrillation (HR=2.669; 95% CI= 2.110-3.377)(9). |
|  | Metabolic syndrome(10) | 1. SO in older men was associated with two-fold increased likelihood for metabolic syndrome (OR=2.07; 95% CI=1.21-3.55)(10). |
|  | Mortality(5, 11-18) | 1. Male patients with SO exhibited the highest mortality risk (HR = 1.41; 95% CI = 1.22-1.63)(11). 2. Risk for death was significantly increased in SO defined by WC (HR=1.19; 95% CI= 1.02-1.38), borderline in the BMI-defined group (HR=1.15; 95% CI= 1.00-1.33), and not significant in the %BF-defined group (HR=1.03; 95% CI= 0.86-1.23)(12). 3. SO (HR=5.23; 95% CI=2.46-11.14) was independently related to all-cause mortality(13). 4. The 75-year-old women with SO had a higher risk (HR= 3.25; 95% CI=1.2-8.9) of dying within 10 years compared to those with a “normal” phenotype(14). 5. SO classified by BF% (HR = 2.94; 95% CI = 1.25-6.92), BMI_25_ (HR =13.38; 95% CI = 2.82-63.48), VFA (HR = 4.60; 95% CI =1.25-16.94), and AF (HR =3.77; 95% CI =1.38-10.28) was significantly associated with mortality(15). 6. The incidence of SO is significantly associated with all-cause mortality (HR = 3.63; 95% CI = 1.52-8.68)(5). 7. SO (HR= 4.85; 95% CI= 1.91-12.31) is independently associated with 5-year mortality in older adults(16). 8. When defining SO based on percentage body fat, the mortality rate in the SO group was significantly higher than that in the non- SO group (HR= 1.97; 95% CI= 1.14-3.38). The survival time in the SO group was significantly shorter than that in the non- SO group(17). 9. SO obesity is significantly associated with mortality in older adults (HR=6.54; 95% CI=1.09-39.38)(18). |
| **Safety-related incidents** | Fracture(15, 19) | 1. In older men, FNIH defined SO men have increased fracture risk over 6 years compared with non-SO men (HR= 2.38; 95% CI=1.29-4.36) (19). 2. SO classified by BF% (HR = 2.94; 95% CI = 1.25-6.92), BMI_25_ (HR =13.38; 95% CI = 2.82-63.48), VFA (HR = 4.60; 95% CI =1.25-16.94), and AF (HR =3.77 , 95% CI =1.38-10.28) was significantly associated with fracture incident (15). |
|  | Fall(3, 19) | 1. EWGSOP-defined SO is associated with increased fall rates over 2 years (IRR=1.66; 95% CI=1.16-2.37)(19). 2. ESPEN-EASO -defined SO was associated with higher 12-month incident fall rates (OR = 2.03; 95% CI =1.23-3.36)(3) |
| **Neuropsychological and Frailty Status** | Depression(20, 21) | 1. The SO is a risk factor for depressive symptoms in older adults (OR=1.79; 95% CI=1.10-2.89). 2. SO predicts non-remission of depression (OR=1.06; 95% CI=1.01-1.11). |
|  | Frailty(2) | SO was significantly associated with frailty (OR=2.00; 95% CI=1.42-2.82). |

**Abbreviations:** 6MWD, 6-minute walk distance; AF, Android fat; BF%, Body fat percentage; BMI, Body mass index; CI, Confidence interval; ESPEN-EASO, European Society for Clinical Nutrition and Metabolism – European Association for the Study of Obesity; EWGSOP, European Working Group on Sarcopenia in Older People; FNIH, Foundation for the National Institutes of Health; HR, Hazard ratio; IRR, Incidence rate ratio; OR, Odds ratio; SPPB, Short Physical Performance Battery; VFA, Visceral fat area; WC, Waist circumference.

**Reference**

1.	Baumgartner RN, Wayne SJ, Waters DL, Janssen I, Gallagher D, Morley JE. Sarcopenic obesity predicts instrumental activities of daily living disability in the elderly. Obesity research. 2004;12(12):1995-2004.

2.	Hirani V, Naganathan V, Blyth F, Le Couteur DG, Seibel MJ, Waite LM, et al. Longitudinal associations between body composition, sarcopenic obesity and outcomes of frailty, disability, institutionalisation and mortality in community-dwelling older men: The Concord Health and Ageing in Men Project. Age &amp; Ageing. 2017;46(3):413-20.

3.	Scott D, Blyth F, Naganathan V, Le Couteur DG, Handelsman DJ, Waite LM, et al. Sarcopenia prevalence and functional outcomes in older men with obesity: Comparing the use of the EWGSOP2 sarcopenia versus ESPEN-EASO sarcopenic obesity consensus definitions. Clin Nutr. 2023;42(9):1610-8.

4.	Yoshimura Y, Wakabayashi H, Nagano F, Matsumoto A, Shimazu S, Shiraishi A, et al. The Applicability of the ESPEN and EASO-Defined Diagnostic Criteria for Sarcopenic Obesity in Japanese Patients after Stroke: Prevalence and Association with Outcomes. Nutrients. 2022;14(19).

5.	Saito H, Matsue Y, Kamiya K, Kagiyama N, Maeda D, Endo Y, et al. Sarcopenic obesity is associated with impaired physical function and mortality in older patients with heart failure: insight from FRAGILE-HF. BMC geriatrics. 2022;22(1):556.

6.	Morikawa M, Lee S, Makino K, Harada K, Katayama O, Tomida K, et al. Sarcopenic Obesity and Risk of Disability in Community-Dwelling Japanese Older Adults: A 5-Year Longitudinal Study. Journal of the American Medical Directors Association. 2023;24(8):1179-.

7.	Stephen WC, Janssen I. Sarcopenic-obesity and cardiovascular disease risk in the elderly. Journal of Nutrition, Health &amp; Aging. 2009;13(5):460-6.

8.	Yoon HJ, Park KW, Seo YH. Association of Sarcopenia and Visceral Obesity with Clinical Outcomes Among Older Adults with Cardiovascular Disease: A Retrospective Cohort Study. Journal of Clinical Medicine. 2025;14(12).

9.	Yu X, Chao J, Wang X, Dun S, Song H, Guo Y, et al. Sarcopenic obesity and the risk of atrial fibrillation in non-diabetic older adults: A prospective cohort study. Clinical Nutrition. 2025;47:282-90.

10.	Scott D, Cumming R, Naganathan V, Blyth F, Le Couteur DG, Handelsman DJ, et al. Associations of sarcopenic obesity with the metabolic syndrome and insulin resistance over five years in older men: The Concord Health and Ageing in Men Project. Experimental Gerontology. 2018;108:99-105.

11.	Atkins JL, Whincup PH, Morris RW, Lennon LT, Papacosta O, Wannamethee SG. Sarcopenic Obesity and Risk of Cardiovascular Disease and Mortality: A Population-Based Cohort Study of Older Men. Journal of the American Geriatrics Society. 2014;62(2):253-60.

12.	Sanada K, Chen R, Willcox B, Ohara T, Wen A, Takenaka C, et al. Association of sarcopenic obesity predicted by anthropometric measurements and 24-y all-cause mortality in elderly men: The Kuakini Honolulu Heart Program. Nutrition. 2018;46:97-102.

13.	Atmis V, Yalcin A, Silay K, Ulutas S, Bahsi R, Turgut T, et al. The relationship between all-cause mortality sarcopenia and sarcopenic obesity among hospitalized older people. Aging Clinical &amp; Experimental Research. 2019;31(11):1563-72.

14.	von Berens Å, Obling SR, Nydahl M, Koochek A, Lissner L, Skoog I, et al. Sarcopenic obesity and associations with mortality in older women and men - a prospective observational study. BMC Geriatrics. 2020;20(1):1-10.

15.	Chuan F, Chen S, Ye X, Kang S, Mei M, Tian W, et al. Sarcopenic obesity predicts negative health outcomes among older patients with type 2 diabetes: The Ageing and Body Composition of Diabetes (ABCD) cohort study. Clinical Nutrition. 2022;41(12):2740-8.

16.	Ulugerger Avci G, Bektan Kanat B, Can G, Suzan V, Unal D, Degirmenci P, et al. The impact of sarcopenia and obesity on mortality of older adults: five years results. Irish Journal of Medical Science. 2023;192(5):2209-16.

17.	Chen XY, Li M, Huang S, Luo SY, Zhang WY, JIang WH, et al. Sarcopenic obesity and mortality risk in nursing home residents: a prospective cohort study based on six obesity indicators. Chinese Journal of Geriatrics. 2025;44(7):943-50.

18.	Zong M, Zhao AD, Han WJ, Chen YQ, Weng TW, Li SJ, et al. Sarcopenia, sarcopenic obesity and the clinical outcome of the older inpatients with COVID-19 infection: a prospective observational study. Bmc Geriatrics. 2024;24(1).

19.	Scott D, Chandrasekara SD, Laslett LL, Cicuttini F, Ebeling PR, Jones G. Associations of Sarcopenic Obesity and Dynapenic Obesity with Bone Mineral Density and Incident Fractures Over 5–10 Years in Community-Dwelling Older Adults. Calcified Tissue International. 2016;99(1):30-42.

20.	Hamer M, Batty GD, Kivimaki M. Sarcopenic obesity and risk of new onset depressive symptoms in older adults: English Longitudinal Study of Ageing. International Journal of Obesity. 2015;39(12):1717-20.

21.	Kokkeler KJE, van den Berg KS, Comijs HC, Oude Voshaar RC, Marijnissen RM. Sarcopenic obesity predicts nonremission of late-life depression. International Journal of Geriatric Psychiatry. 2019;34(8):1226-34.
